# Exploring familial factors in the migrant mortality advantage among domestic migrants in later life: Zeeland, the Netherlands, 1812–1962^☆^

**DOI:** 10.1016/j.ssmph.2023.101359

**Published:** 2023-02-09

**Authors:** Rick J. Mourits, Paul Puschmann

**Affiliations:** a International Institute of Social History, Cruquiusweg 31, 1019 AT, PO Box 2169, 1000 CD, Amsterdam, the Netherlands; b Department of Economic, Social, and Demographic History, Radboud University, Erasmusplein 1, 6525 HT, PO Box 9102, 6500 HC, Nijmegen, the Netherlands

**Keywords:** Migration, Healthy immigrant paradox, Migrant mortality advantage, Healthy migrant effect, Family effects, Mortality

## Abstract

Many historical and contemporary studies have shown that migrants enjoy survival advantages over non-migrants, even if they originate from higher mortality regimes and have a lower socio-economic and educational status compared to non-migrants in the destination area. This so-called migrant mortality advantage or healthy migrant effect is explained in various ways. One of the main explanations refers to selection effects in the area of origin in the sense that healthier individuals are more likely to move compared to less healthy individuals. So far life-course analysis on the healthy migrant effect were focused on the survival chances of individual migrants compared to non-migrants. However, kin members of migrants might also enjoy survival advantages, given that health and mortality are often clustered in families due to shared environments, behaviors, resources, and household dynamics. We study whether kin members of migrants within the Dutch province of Zeeland also enjoy survival advantages. Although we find a mortality advantage for the migrating men and women in our sample, we find no mortality advantage for their siblings or offspring. However, there was a gendered effect of familial migration in the sense that women, contrary to men, had higher mortality risks if their siblings and parents migrated.

## Highlights

• Multiple studies show that migrants enjoy survival advantages over non-migrants.
• We study if internal migrants and their family members had similar survival chances.
• We find a survival advantage for migrants, but not for their siblings or offspring.
• However, sisters and daughters of migrants had consistently higher mortality rates.
• More studies on migrant family networks are needed to understand the gender effect.

## 1 Introduction

Migration is not for the faint of heart. Moving from A to B generally means leaving a social network behind, crossing physical and mental borders, and dealing with the stress of living in a new and unfamiliar environment. Cross-cultural migrants also have to learn a new language, deal with the challenges of obtaining visa and residence permits, and they have to acculturate, and get familiar with the laws, regulations and conventions in the destination society. Moreover, migrants might face prejudice, discrimination, and bureaucratic challenges, including the recognition of credentials. Migration studies show that migrants – both in the past and today – often face social exclusion in key domains of the destination society. Migrants from low income countries with a low educational status have a higher risk of ending up in poorly paid and unhealthy jobs, they are more likely to live in poor and less healthy neighborhoods, and have often only limited access to social protection. Last but not least, cross-cultural differences, institutional obstacles and language difficulties might hinder migrants from getting optimal health care (Fangen, Fossan & Mohn, 2016; Gil-González et al., 2015).

Given the stress and difficulties migrants face, one would expect that migrants pay a health price for their move. However, ever since the 1980’s a multitude of studies have consistently shown that migrants enjoy survival advantages over non-migrants, and that this survival advantage – also known as “healthy immigrant paradox”, “migrant mortality advantage”, or “healthy migrant effect” – occurs in many different contexts, and among various migrant groups. It has, for example, been documented for Mexican migrants in the US (Lariscy et al., 2015; Markides & Coreil, 1986; Markides & Eschbach, 2005), MENA migrants in Europe (Deboosere & Gadeyne, 2005; Khlat & Courbage, 1996), as well as for various historical migrant populations (Alter & Oris, 2005; Khlat & Courbage, 1996; Oris & Alter, 2001; Puschmann et al., 2016).

Various earlier studies have demonstrated that the migration mortality advantage is not some kind of statistical artefact and cannot – or only partially – be explained by selective return migration of the frail and sick, as suggested by the Salmon-bias hypothesis (Puschmann, Donrovich & Matthijs 2017; Razum et al., 2000; Van den Berg et al., 2020; Wallace & Kulu, 2014). In order to explain the observed mortality advantage among migrants, scholars have suggested that a selection effect is at work. However, the suggested selection mechanism behind the healthy migrant effect remains unclear and lacks theoretical underpinning. So far life-course analysis on the healthy migrant effect were focused on the survival chances of individual migrants compared to non-migrants. Generally, it is thought that the healthiest individuals in society migrate. After all, it requires strength and perseverance to reach and succeed in far destinations. Not surprisingly, survival advantages of migrants are stronger the further, and the more often migrants move (Puschmann et al., 2016; Puschmann et al., 2017). This has been interpreted as better health enables people to migrate further and more frequently. However, it remains to be seen whether the selection mechanism behind the healthy migrant effect operates at the individual level, the family level, or a combination of both. After all health and its determinants are correlated among family members, as siblings share environments, behavior, socioeconomic resources, and genetic dispositions that can create health advantages or disadvantages early in life with long-lasting effects across the life course (Donrovich, Puschmann & Matthijs, 2014; Mourits et al., 2020; Van Dijk, Janssens, & Smith, 2019). Furthermore, parents and siblings can transmit health protective behaviors by ways of socialization processes.

Relatively little studies have investigated the healthy migrant effect from a family perspective, and those studies that exist focus on the survival chances of the children of migrants (see e.g. Hajat et al., 2010; Wallace & Kulu, 2015). Most of these studies found no health advantage among the offspring of migrants over the life course, indicating that healthy migrant effects are not transferred between generations. Yet, they might have been looking in the wrong place for familial effects. Migrant mortality advantages are probably rooted in the family of origin, where non-migrating siblings might enjoy survival advantages as well. Since most studies focus on international migrants and only feature data on the receiving society, and thus lack data on the sending society, these questions are hard to address for many contemporary societies. The focus on international migration in contemporary literature makes it hard to study effects from the sending region. Therefore, the most important insights on familial influences on the migrant mortality advantage stem from historical studies that focused on internal migration.

Historical studies have been able to study the life courses of domestic migrants and highlighted the importance of familial effects. In a study on nineteenth-century east Belgium, Oris and Alter (2001) showed that unmarried individuals between the ages of 15 and 29 were less likely to out-migrate after a recent death in the household. Evidence from the Netherlands shows that migration experiences of family members and acquaintances resemble one another, as these migration experiences create social networks that determine how and whether people moved (Kok, Mandemakers & Mönkediek, 2014). The question then is whether children and siblings of migrants also enjoy a similar mortality advantage. If this is the case, this suggests that selection of healthy migrants occurs not only at the individual, but also at the family level. If we do not find such an effect, we may tentatively assume that the migrant mortality advantage is mainly a selection effect at the individual level and “healthy migrants” are selected at random from a sending society.

In this paper, we explore whether the “migrant mortality advantage” is due to a selection effect at the familial level or rather goes back to a selection effect at the individual level. To answer this question, we enquire whether siblings and children of migrants had survival advantages over individuals without a migrating sibling or parent, and explore whether the migration status of parents or siblings is a predictor of the survival chances of an individual’s survival chances. For this purpose, we use reconstituted family data from the historical dataset LINKS-Zeeland (Mourits et al., 2022) to study 8030 disjoint families with information on 16,060 parents and 25,538 children with a known sibling over age 50. We specifically look at intra-regional migration, so that we can compare migrants with stayers and keep differences between the sending and receiving population as small as possible. These intraprovincial migrants had a similar survival advantage over non-migrants as individuals who migrated to other provinces in the Netherlands (Van den Berg et al., 2020). We use Cox proportional hazard models to compare the survival of: 1) individuals from families with no migration, 2) individuals from families in which a sibling migrated, and 3) individuals from families of which a parent migrated. We distinguish between individuals who moved and individuals who remained in their place of birth over the life course. Thereupon, we conclude whether the healthy migrant effect is mainly due to familial or individual selection effects and discuss what this outcomes means for enquiries into migration.

## 2 Data & methods

We use LINKS-Zeeland (Mourits et al., 2022; Mourits, van Dijk, & Mandemakers, 2020) to study how familial migration histories affect individual survival. LINKS-Zeeland is a dataset that contains demographic information on family relations and life courses for individuals who lived in the Dutch province of Zeeland during the 19th and early-20th centuries. The life course reconstructions are based on the births, marriages, and deaths that were registered by the local municipalities within three days after the event took place. Registration was highly standardized and accepted by the local populace, so that practically all births, marriages, and deaths were registered. Moreover, practically all registrations have survived the test of time. Civil certificates were made in duplicate and stored at separate locations, which protected the civil registry against the fires and floods that damaged parts of the Zeeland population registers.

**Fig. 1:**
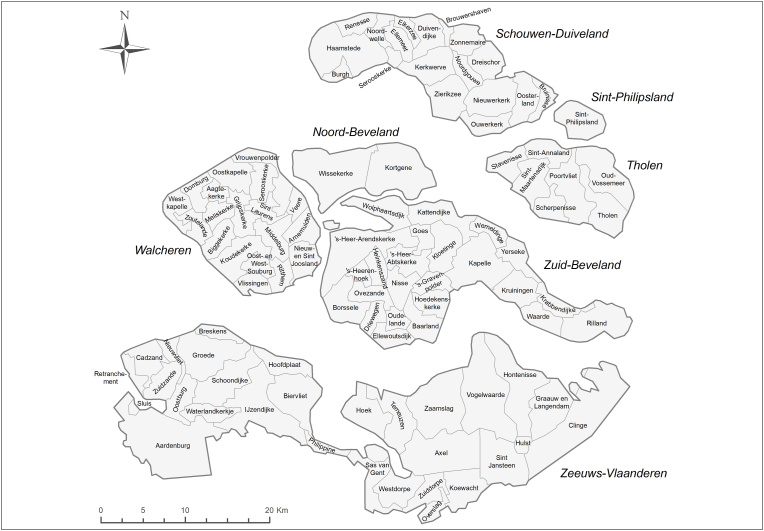
Islands and municipalities in Zeeland.

Source: Hermsen, 2018.

Fig. 1 shows a map of 19th-century Zeeland. The province is a largely rural island archipelago in the southwest of the Netherlands that did not strongly urbanize or industrialize. During our research period it consisted of the islands Schouwen-Duiveland, Sint-Philipsland, Tholen, Noord-Beveland, and Walcheren, the peninsula of Zuid-Beveland, and the mainland of Zeeuws-Vlaanderen bordering Belgium in the south. Zeeland was a commercial-agricultural region producing livestock, beans, beets, grain, madder, and potatoes (Priester, 1998, p. 216). A sizeable contingent of the population also worked in transport and trade, mainly as freighters or petty merchants (Wintle, 1992). Walcheren was home to the only two cities in Zeeland, although every island had one or multiple larger towns that functioned as gateways to the world. People mainly moved for marriage, to find work, or receive familial support (Kok, 1997). The province was economically saturated, which meant that many (agricultural) laborers left the province to find employment elsewhere, especially as agriculture mechanized (Wintle, 1992). About half the population stayed in their municipality of birth (Van den Berg et al., 2020). Migrants either moved to a rural village on the same island, the larger towns in Zeeland, or left for the rapidly growing city of Rotterdam or the promise of land in the New World (Bras, 2002, p. 100; Wintle, 1992).

### 2.1 Sample selection

LINKS is especially suited to follow families over time, even though we do not follow individuals continuously over time and cannot trace each individual migration movement over the life course (see e.g. Fedorova et al., 2022; Sesma Carlos et al., 2022). An explorative study by Van den Berg et al. (2020) has shown that health advantages due to migration can still be reliably estimated with civil registry data, even though we cannot distinguish between stayers and return migrants. The family and life course reconstructions in LINKS are of the same quality as historical sources that follow individuals actively over time, so lang as an individual’s birth, marriage, and death are observed in the data base. As a result, we can study individuals who never left the province of Zeeland, as they were at risk of experiencing birth, marriage, and deaths in the Zeeland civil registers, whereas we cannot estimate reliable statistics for individuals who migrated out of Zeeland. Therefore, we study parents and children who lived their entire lives in Zeeland to ascertain the validity of our results.

An overview of the selection process and the resulting sample is given in Table 1. First, we select complete families. Only those parents were selected of whom it could be assumed that they lived their whole life in Zeeland. This allows us to compare stayers and domestic migrants within Zeeland. Sibships are reconstructed through the parental marriage certificate and can be deemed complete if both parents had a known death certificate. Therefore, we selected families of which the parents had a known place of birth, available marital information, and a death certificate. Second, we select offspring who died after age 50, as these individuals lived their whole lives in Zeeland and were not at risk of migrating out of the province (Fedorova et al., 2022; Kok, 1997). To be able to study the effects of sibling migration, these individuals needed to have a known place of birth and death as well as a sibling who lived until 50. Finally, disjoint family trees were selected to prevent that certain families were overrepresented in our data. We made sure that offspring – the daughters and sons in our sample – did not return as parents and excluded half-sibs from the data by randomly selecting one childbearing family if fathers or mothers reproduced with more than one marital partner. This left us with a sample of 16,060 parents and 25,538 children with a known sibling over age 50, briefly described in Table 2.

**Table 1:** Sampling procedure.

	Parents	Offspring
Selections parents		
Married between 1812 and 1862	113,905	–
Known to have had children	87,587	254,281
Known death certificate	63,205	225,770
Known death certificate for at least one partner	51,932	166,238
Known places of birth and death	48,384	147,645

**Selections offspring**		
Lived past age 50	34,473	43,661
Known places of birth and death	34,242	43,051
At least 1 known sibling	22,307	36,753

**Disjoint families**		
Children of first known generation	16,205	25,951
No half siblings	16,060	25,538

**Table 2:** Characteristics of parents and offspring in the studied sample of LINKS Zeeland.

	Father	Mothers	Sons	Daughters
Sample				
N	8030	8030	12,726	12,822
Birth cohorts	1755–1843	1773–1843	1812–1885	1812–1884
Migration				
*-Stayer*	4546 (56.6%)	4064 (50.6%)	7140 (56.9%)	6376 (49.7%)
*-Migrated*	3448 (43.4%)	3974 (49.4%)	5586 (43.1%)	6446 (50.3%)

**Demographic indicators**				
Mean age at death^a^	65.0 (15.1)	65.8 (16.3)	73.2 (10.6)	73.7 (10.6)
Mode age at death	75	76	75	77
Mean sibship size	–	–	8.1 (3.4)	8.2 (3.4)

a Mean + standard deviation between brackets.

### 2.2 Measurement

Migration is measured by comparing the municipality of birth and the municipality of death. In total, 45% of all fathers and sons in our sample migrated within Zeeland, whereas 50% of all women in our sample migrated within the province. These numbers are in line with an earlier study on the female Zeeland population (Bras, 2002, p. 129). Sibling migration is measured as the total number of siblings that died outside their municipality of birth, excluding ego. Parental migration was measured in four different groups: 1) no parent migrated, 2) father migrated, mother stayed in municipality of birth, 3) mother migrated, father stayed in municipality of birth, and 4) both parents migrated.

Furthermore, we include sex, birth year, sibship size, marital status, and social class. Year of birth is measured linearly. Sibship size is measured in three categories 2–4, 5–8, and 9+. Socioeconomic class is measured as the highest social position before age 50, split into six categories. The elite were those who performed learned professions, such as artists, clergymen, doctors, engineers, lawyers, pharmacists, teachers, and veterinarians. Middle strata encompass proprietors, managers, clerks, salesmen, and craftsmen. Laborers are all those who performed semi- or unskilled labor outside agriculture. Farmers comprise farmers. Farm laborers are those who performed semi- or unskilled farm labor. Finally, we include individuals for whom no occupation was known. For women, these are likely random missings as female occupations were generally poorly recorded (Boter & Woltjer, 2020; Walhout & van Poppel, 2003). For men, we might be dealing with informed missings, as occupations were recorded well and only the affluent could afford to be idle for prolonged periods of time.

Environmental variables include urbanization, disease load, demographic pressure, and island of birth. Urbanization is measured as the logarithm of the number of inhabitants. Disease load is measured as the level of child mortality, that is the percentage of newborns who died before their fifth birthday. Demographic pressure is estimated by the net migration rate, as steady out-migration was a way to cope with the economic stagnation that occurred in Zeeland. Included regions of birth are the islands Schouwen-Duiveland, Sint Philipsland, Tholen, Walcheren, Noord-Beveland, and Zuid-Beveland as well as the mainland of Zeeuws-Vlaanderen, which is connected to Belgium.

### 2.3 Statistics

The survival of the offspring in our data was studied using Cox regressions. Analyses were done using R version 4.1.3 and the survival and coxme packages (R Core Team, 2022; Therneau, 2022a, 2022b. To deal with robustness issues, we censored our analyses at age 100. Associations between migration and mortality after age 50 are estimated separately for men and women, as migration is thought to affect both sexes differently. For both sexes, we estimate three different models to study the association between (familial) migration and individual later-life mortality. These models are defined as:

$$\lambda \left(t_{j}\right)=\lambda_{0}\left(t_{j}\right)\exp\left(\beta_{z}Z_{j}+\beta_{x}X_{j}+\beta_{a}A_{j}+\beta_{a}B_{j}\right)$$

*t*_*j*_ is the age at death or the age at last observation for child *j*. $\lambda_{0}\left(t_{j}\right)$ refers to the baseline hazard, which is left unspecified. $\beta_{z}$ is a vector of regression coefficients for the main effect (Z) which corresponds to whether someone migrated. $\beta_{x}$ contains the regression effects of a set of familial migration variables (X). We estimate models without familial migration (model 1), sibling migration (model 2), and parental migration (model 3). $\beta_{a}$ contains the regression effects of individual-level covariates of year of birth, sibship size, and social class (A), whereas $\beta_{b}$ contains the regression effects of environmental variables child mortality rate, number of inhabitants, net migration rate, and island of birth (B). The main effect (Z) and covariates (A) and (B) are present in every model, whereas the set of variables (X) differs per model.

#### 2.3.1 Non-proportionality

Non-proportional hazard ratios – that is hazard ratios that differed by age – were estimated using step functions (Therneau et al., 2022). For the proportional hazard ratios these models return only one hazard ratio, whereas for the non-proportional effects a hazard ratio was estimated per 10-year age interval: 50–59, 60–69, 70–79, etc. These implicit interactions with time are shown in the table footnotes and indicate how effect sizes change with age.

#### 2.3.2 Robustness checks

To test for unobserved similarities between siblings, we also estimated frailty models as a robustness check. These frailty models were defined as:

$$\lambda \left(t_{ij}\right)=u_{i}\lambda_{0}\left(t_{ij}\right)\exp\left(\beta_{z}Z_{ij}+\beta_{x}X_{ij}+\beta_{a}A_{ij}+\beta_{a}B_{j}\right)$$

These models are very similar to the standard Cox proportional hazard models. However, *t*_*ij*_ is the age at death or the age at last observation for child *j* in family *i*. *u* > 0 refers to an unobserved random effect (frailty) shared by children of a given family. This unobserved heterogeneity shared within sibships was assumed to follow a log-normal distribution. The outcomes of these robustness checks are shown in supplementary tables A1 and A2.

We also control whether an individual’s own migration status interacted with the familial background of migration. Therefore, we check whether the associations between familial migration and individual later-life mortality in models 2 and 3 are different for individual migrants and stayers. These models are defined as:

$$\lambda \left(t_{j}\right)=\lambda_{0}\left(t_{j}\right)\exp\left(\beta_{x}X_{j}+\beta_{a}A_{j}+\beta_{a}B_{j}\right)$$

and are a small variation on the initial step models. The only difference is that we removed the regression coefficients for whether someone migrated, $\beta_{z}Z_{j}$, as effects for migrants and stayers are now estimated separately. The outcomes of these robustness checks are shown in supplementary tables A3 to A6.

## 3 Results

In our tables, effect sizes will be reported as Exp(β), i.e. Hazard Ratio’s (HR). We further report 95% confidence intervals and p-values. Non-proportional effects are indicated with an ^a^, results of the step functions are shown in the table’s footnotes. In the text, we discuss the HRs and 95% confidence intervals in terms of survival advantages. A HR of 0.92, for example, indicates a 8% lower chance of dying and from here we will refer to this as a survival advantage of 8%.

### 3.1 Individual migration

Table 3 shows that for both men and women, there was a migrant survival advantage after age 50. Men who migrated within Zeeland had a survival advantage of 8% (HR: 0.92, CI: 0.89–0.96) over men who stayed in their municipality of birth. Migrating women had a smaller survival advantage over their non-migrating peers than migrating men. Migrant women had a survival advantage of 6% (HR 0.94, CI: 0.91–0.98) over women who stayed in their municipality of birth. This female “migrant mortality advantage” is even smaller after we control for non-linearity, as female migrants only have a survival advantage of 4% (HR: 0.96, CI: 0.93–1.00) over female stayers after age 60. These effects are similar after controlling for unobserved familial effects in tables A1 and A2. This indicates that there is a “migrant mortality advantage” for both men and women, but that the effect is more prominent for men, suggesting that male migration is more selective in terms of good health.

**Table 3:** Association between individual migration and later-life survival.

	Men				Women			
	N_offspring_	N_families_	HR + 95% CI	p-value	N_offspring_	N_families_	HR + 95% CI	p-value
Migration								
Ego migrated								
⁃ *No*	*7135*	*4859*	ref.	ref.	*6377*	*4304*	ref.	ref.
⁃ *Yes* b	*5584*	*3759*	**0.92 (0.89–0.96)**	**<0.001**	*6441*	*4198*	**0.94 (0.91–0.98)**	**<0.001**

**Control variables**								
Year of birth^a^	*12,719*	*6746*	**0.95 (0.94–0.96)**	**<0.001**	*12,818*	*6867*	**0.98 (0.96–0.99)**	**<0.001**
Sibship size								
⁃ *2-4*	*1308*	*912*	ref.	ref.	*1319*	*919*	ref.	ref.
⁃ *5-8*	*4955*	*2836*	1.02 (0.96–1.09)	0.486	*4980*	*2889*	0.98 (0.93–1.05)	0.599
⁃ *9+*	*6456*	*2998*	1.04 (0.98–1.10)	0.196	*6519*	*3059*	1.02 (0.96–1.08)	0.508
Social class								
⁃ *Elite*	*271*	*246*	**1.20 (1.06–1.36)**	**0.005**	*39*	*39*	0.93 (0.68–1.23)	0.969
⁃ *Middle class*	*1397*	*1169*	1.00 (0.94–1.07)	0.971	*279*	*254*	0.98 (0.90–1.06)	0.523
⁃ *Skilled laborers*	*2473*	*1846*	0.96 (0.91–1.02)	<0.159	*291*	*264*	**0.87 (0.78–0.97)**	**0.012**
⁃ *Laborers*	*2482*	*1877*	ref.	ref.	*5498*	*3709*	ref.	ref.
⁃ *Farmers*	*1933*	*1288*	**0.90 (0.85–0.96)**	**<0.001**	*668*	*548*	0.99 (0.90–1.03)	0.238
⁃ *Farm workers*	*2221*	*1693*	0.95 (0.90–1.01)	0.091	*526*	*452*	1.01 (0.94–1.12)	0.541
⁃ *Unknown*	*1942*	*1633*	**1.08 (1.02–1.15)**	**0.010**	*5517*	*3654*	1.00 (0.93–1.01)	0.118

**Environment**								
Child mortality rate	*12,719*	*6746*	0.99 (0.97–1.01)	0.292	*12,818*	*6867*	1.00 (0.98–1.02)	0.982
Number of inhabitants	*12,719*	*6746*	1.01 (0.98–1.05)	0.509	*12,818*	*6867*	1.03 (0.99–1.07)	0.103
Net migration rate	*12,719*	*6746*	0.98 (0.95–1.02)	0.413	*12,818*	*6867*	1.03 (1.00–1.07)	0.091
Island of birth								
⁃ *Noord-Beveland*	542	*300*	ref.	ref.	*533*	*291*	ref.	ref.
⁃ *Schouwen-Duiveland*	*1614*	*869*	0.98 (0.89–1.09)	0.734	*1583*	*879*	0.97 (0.88–1.08)	0.585
⁃ *Sint Philipsland*	100	*46*	0.81 (0.66–1.01)	0.058	*88*	*48*	**0.77 (0.62–0.97)**	**0.027**
⁃ *Tholen*	*1238*	*638*	0.91 (0.82–1.01)	0.065	*1,15*	*628*	0.98 (0.88–1.08)	0.662
⁃ *Walcheren*	*3179*	*1681*	1.00 (0.91–1.09)	0.954	*3302*	*1744*	1.05 (0.96–1.16)	0.271
⁃ *Zeeuws Vlaanderen*	*3375*	*1840*	1.00 (0.91–1.10)	0.990	*3537*	*1883*	1.02 (0.93–1.12)	0.655
⁃ *Zuid-Beveland*	*2671*	*1430*	1.06 (0.96–1.16)	0.239	*2670*	*1463*	1.06 (0.97–1.17)	0.199

a The survival advantage for year of birth decreased with age from 0.91 (CI: 0.88–0.94) to 0.94 (CI: 0.91–0.96), 0.95 (CI: 0.93–0.97), 0.98 (CI: 0.96–1.01), and 0.95 (CI: 0.90–1.02).

b Associations with migration decreased from 0.81 (CI: 0.74–0.90) before age 60 to 0.96 (CI: 0.93–1.00) between ages 60–100. The survival advantage for year of birth decreased linearly with age from 0.93 (CI: 0.90–0.96) to 0.96 (CI: 0.93–0.98), 0.98 (CI: 0.96–1.00), 1.00 (CI: 0.98–1.03), and 1.04 (CI: 0.98–1.10).

### 3.2 Sibling migration

Table 4 shows that men with two or more migrating siblings had a small, but non-significant survival advantage. The survival advantage was 3–4% for men with 2 migrant siblings (HR: 0.96, CI: 0.91–1.01), 3 migrant siblings (HR: 0.96, CI: 0.90–1.02), 4 migrant siblings (HR: 0.97, CI: 0.89–1.05), or 5 migrant siblings (HR: 0.96, CI: 0.86–1.06) and only 2% (HR: 0.98, CI: 0.89–1.09) for men with 6 or more migrating siblings. These effects are similar after controlling for unobserved familial effects in tables A1 and A2 or for interactions between the individual migration status and the familial background of migration in table A3.

**Table 4:** Association between sibling migration and later-life survival.

	Men				Women			
	N_offspring_	N_families_	HR + 95% CI	p-value	N_offspring_	N_families_	HR + 95% CI	p-value
Migration								
Ego migrated								
⁃ *No*	*7135*	*4859*	ref.	ref.	*6377*	*4304*	ref.	ref.
⁃ *Yes* b	*5584*	*3759*	**0.93 (0.89–0.97)**	**<0.001**	*6441*	*4198*	**0.92 (0.89–0.96)**	**<0.001**
Sibling migration								
⁃ *0*	*3601*	*2416*	ref.	ref.	*3602*	*2566*	ref.	ref.
⁃ *1*	*3451*	*2472*	1.01 (0.96–1.06)	0.706	*3511*	*2529*	**1.08 (1.03–1.14)**	**<0.001**
⁃ *2*	*2337*	*1600*	0.96 (0.91–1.01)	0.118	*2362*	*1580*	**1.08 (1.02–1.14)**	**0.009**
⁃ *3*	*1493*	*936*	0.96 (0.90–1.02)	0.213	*1537*	*915*	**1.12 (1.05–1.19)**	**<0.001**
⁃ *4*	908	*503*	0.97 (0.89–1.05)	0.406	*904*	*505*	**1.08 (1.00–1.17)**	**0.044**
⁃ *5*	416	*201*	0.96 (0.86–1.06)	0.416	*401*	*205*	**1.16 (1.04–1.29)**	**0.007**
⁃ *6+*	513	*181*	0.98 (0.89–1.09)	0.765	*501*	*167*	**1.11 (1.00–1.22)**	**0.049**

**Control variables**								
Year of birth^a^	*12,719*	*6746*	**0.95 (0.94–0.96)**	**<0.001**	*12,818*	*6867*	**0.98 (0.96–0.99)**	**<0.001**
Sibship size								
⁃ *2-4*	*1308*	*912*	ref.	ref.	*1319*	*919*	ref.	ref.
⁃ *5-8*	*4955*	*2836*	1.03 (0.97–1.10)	0.306	*4980*	*2889*	0.97 (0.91–1.03)	0.334
⁃ *9+*	*6456*	*2998*	1.06 (0.99–1.13)	0.078	*6519*	*3059*	0.99 (0.93–1.06)	0.840
Social class								
⁃ *Elite*	271	*246*	**1.20 (1.05–1.36)**	**0.005**	*39*	*39*	0.93 (0.68–1.27)	0.642
⁃ *Middle class*	*1397*	*1169*	1.00 (0.94–1.07)	0.999	*279*	*254*	0.98 (0.87–1.11)	0.784
⁃ *Skilled laborers*	*2473*	*1846*	0.96 (0.91–1.02)	0.157	*291*	*264*	**0.88 (0.78–0.99)**	**0.030**
⁃ *Laborers*	*2482*	*1877*	ref.	ref.	*5498*	*3709*	ref.	ref.
⁃ *Farmers*	*1933*	*1288*	**0.91 (0.85–0.96)**	**0.002**	*668*	*548*	0.99 (0.91–1.02)	0.718
⁃ *Farm workers*	*2221*	*1693*	0.95 (0.90–1.01)	0.107	*526*	*452*	1.00 (0.91–1.10)	0.994
⁃ *Unknown*	*1942*	*1633*	**1.08 (1.02–1.15)**	**0.009**	*5517*	*3654*	1.00 (0.96–1.04)	0.890

**Environment**								
Child mortality rate	*12,719*	*6746*	0.99 (0.97–1.01)	0.302	*12,818*	*6867*	1.00 (0.98–1.02)	0.983
Number of inhabitants	*12,719*	*6746*	1.01 (0.97–1.05)	0.645	*12,818*	*6867*	**1.04 (1.01–1.08)**	**0.025**
Net migration rate	*12,719*	*6746*	0.98 (0.95–1.02)	0.408	*12,818*	*6867*	1.04 (1.00–1.07)	0.060
Island of birth								
⁃ *Noord-Beveland*	542	*300*	ref.	ref.	*533*	*291*	ref.	ref.
⁃ *Schouwen-Duiveland*	*1614*	*869*	0.99 (0.89–1.09)	0.807	*1583*	*879*	0.97 (0.87–1.07)	0.552
⁃ *Sint Philipsland*	100	*46*	0.81 (0.66–1.01)	0.060	*88*	*48*	**0.79 (0.63–0.99)**	**0.043**
⁃ *Tholen*	*1238*	*638*	0.91 (0.82–1.01)	0.067	*1,15*	*628*	0.98 (0.88–1.08)	0.643
⁃ *Walcheren*	*3179*	*1681*	1.01 (0.92–1.10)	0.911	*3302*	*1744*	1.04 (0.94–1.14)	0.471
⁃ *Zeeuws Vlaanderen*	*3375*	*1840*	1.01 (0.92–1.10)	0.090	*3537*	*1883*	1.01 (0.92–1.11)	0.791
⁃ *Zuid-Beveland*	*2671*	*1430*	1.06 (0.97–1.17)	0.210	*2670*	*1463*	1.06 (0.96–1.16)	0.255

a The survival advantage for year of birth decreased with age from 0.91 (CI: 0.88–0.94) to 0.94 (CI: 0.91–0.96), 0.95 (CI: 0.93–0.97), 0.98 (CI: 0.96–1.01), and 0.95 (CI: 0.90–1.02).

b Associations with migration decreased from 0.80 (CI: 0.73–0.89) before age 60 to 0.95 (CI: 0.91–0.99) between ages 60–100. The survival advantage for year of birth decreased linearly with age from 0.93 (CI: 0.90–0.96) to 0.95 (CI: 0.93–0.98), 0.98 (CI: 0.96–1.00), 1.00 (CI: 0.98–1.03), and 1.04 (CI: 0.98–1.10).

For women, reverse effects were found. There was a significant survival disadvantage of 8–16% for women with 1 migrating sibling (HR: 1.08, CI: 1.03–1.14), 2 migrating siblings (HR: 1.08, CI: 1.02–1.14), 3 migrating siblings (HR: 1.12, CI: 1.05–1.19), 4 migrating siblings (HR: 1.08, CI: 1.00–1.17), 5 migrating siblings (HR: 1.16, CI: 1.04–1.29), or 6 migrating siblings (HR: 1.11, CI: 1.00–1.22). These effects are similar after controlling for unobserved familial effects in tables A1 and A2 or for interactions between the individual migration status and the familial background of migration in table A4.

For both men and women, the hazard ratios of sibling migration are not dependent on the number of migrating siblings, which indicates that the “migrant mortality advantage” is probably not affected by stacked resources. The adverse relation indicates that female family members of migrants are disadvantaged.

### 3.3 Parental migration

Finally, Table 5 shows that there was almost no difference between men without a migrating parent and men with a migrating father (HR: 1.02, CI: 0.97–1.08), migrating mother (HR: 0.99, CI: 0.94–1.04), or two migrating parents (HR: 1.02, CI: 0.96–1.07). These effects are similar after controlling for unobserved familial effects in tables A1 and A2 or for interactions between the individual migration status and the familial background of migration in table A5.

**Table 5:** Association between parental migration and later-life survival.

	Men				Women			
	N_offspring_	N_families_	HR + 95% CI	p-value	N_offspring_	N_families_	HR + 95% CI	p-value
Migration								
Ego migrated								
⁃ *No*	*7135*	*4859*	ref.	ref.	*6377*	*4304*	ref.	ref.
⁃ *Yes* b	*5584*	*3759*	**0.92 (0.88–0.95)**	**<0.001**	*6441*	*4198*	**0.93 (0.90–0.97)**	**<0.001**
Parental migration								
⁃ *None migrated*	*4215*	*2238*	ref.	ref.	*4219*	*2266*	ref.	ref.
⁃ *Father migrated*	*2185*	*1175*	1.02 (0.97–1.08)	0.364	*2223*	*1194*	**1.06 (1.01–1.12)**	**0.029**
⁃ *Mother migrated*	*2972*	*1572*	0.99 (0.94–1.04)	0.646	*2990*	*1621*	1.04 (0.99–1.09)	0.087
⁃ *Both parents migrated*	*3347*	*1761*	1.02 (0.96–1.07)	0.425	*3386*	*1786*	**1.08 (1.03–1.13)**	**0.001**

**Control variables**								
Year of birth^a^	*12,719*	*6746*	**0.95 (0.94–0.96)**	**<0.001**	*12,818*	*6867*	**0.98 (0.96–0.99)**	**<0.001**
Sibship size								
⁃ *2-4*	*1308*	*912*	ref.	ref.	*1319*	*919*	ref.	ref.
⁃ *5-8*	*4955*	*2836*	1.02 (0.96–1.09)	0.497	*4980*	*2889*	0.99 (0.93–1.05)	0.633
⁃ *9+*	*6456*	*2998*	1.04 (0.98–1.10)	0.204	*6519*	*3059*	1.02 (0.96–1.09)	0.436
Social class								
⁃ *Elite*	*271*	*246*	**1.20 (1.06–1.36)**	**0.005**	*39*	*39*	0.93 (0.68–1.28)	0.656
⁃ *Middle class*	*1397*	*1169*	1.00 (0.94–1.07)	0.965	*279*	*254*	0.98 (0.87–1.11)	0.736
⁃ *Skilled laborers*	*2473*	*1846*	0.96 (0.91–1.02)	0.170	*291*	*264*	**0.87 (0.77–0.98)**	**0.023**
⁃ *Laborers*	*2482*	*1877*	ref.	ref.	*5498*	*3709*	ref.	ref.
⁃ *Farmers*	*1933*	*1288*	**0.90 (0.85–0.96)**	**0.001**	*668*	*548*	0.99 (0.92–1.08)	0.858
⁃ *Farm workers*	*2221*	*1693*	0.95 (0.90–1.01)	0.090	*526*	*452*	1.01 (0.92–1.10)	0.906
⁃ *Unknown*	*1942*	*1633*	**1.08 (1.02–1.15)**	**0.010**	*5517*	*3654*	1.00 (0.96–1.04)	0.969

**Environment**								
Child mortality rate	*12,719*	*6746*	0.99 (0.97–1.01)	0.283	*12,818*	*6867*	1.00 (0.98–1.02)	0.984
Number of inhabitants	*12,719*	*6746*	1.01 (0.98–1.05)	0.501	*12,818*	*6867*	1.03 (1.00–1.07)	0.076
Net migration rate	*12,719*	*6746*	0.98 (0.95–1.02)	0.408	*12,818*	*6867*	1.03 (1.00–1.07)	0.080
Island of birth								
⁃ *Noord-Beveland*	542	*300*	ref.	ref.	*533*	*291*	ref.	ref.
⁃ *Schouwen-Duiveland*	*1614*	*869*	0.98 (0.89–1.11)	0.734	*1583*	*879*	0.96 (0.86–1.06)	0.414
⁃ *Sint Philipsland*	100	*46*	0.82 (0.66–1.01)	0.064	*88*	*48*	**0.79 (0.63–0.99)**	**0.041**
⁃ *Tholen*	*1238*	*638*	0.91 (0.82–1.01)	0.068	*1,15*	*628*	0.97 (0.88–1.08)	0.630
⁃ *Walcheren*	*3179*	*1681*	1.00 (0.91–1.09)	0.941	*3302*	*1744*	1.04 (0.95–1.14)	0.435
⁃ *Zeeuws Vlaanderen*	*3375*	*1840*	1.00 (0.91–1.09)	0.984	*3537*	*1883*	1.01 (0.92–1.11)	0.781
⁃ *Zuid-Beveland*	*2671*	*1430*	1.06 (0.96–1.16)	0.258	*2670*	*1463*	1.05 (0.95–1.15)	0.330

a The survival advantage for year of birth decreased with age from 0.91 (CI: 0.88–0.94) to 0.94 (CI: 0.91–0.96), 0.95 (CI: 0.93–0.97), 0.98 (CI: 0.96–1.01), and 0.95 (CI: 0.90–1.02).

b Associations with migration decreased from 0.80 (CI: 0.73–0.89) before age 60 to 0.96 (CI: 0.92–0.99) between ages 60–100. The survival advantage for year of birth decreased linearly with age from 0.93 (CI: 0.90–0.96) to 0.96 (CI: 0.93–0.98), 0.98 (CI: 0.96–1.00), 1.00 (CI: 0.98–1.03), and 1.04 (CI: 0.98–1.10).

For women, having a migrating parent increased mortality. In comparison to women without a migrating parent, women with a migrating father had a mortality disadvantage of 6% (HR: 1.06, CI: 1.01–1.12), whereas women with a migrating mother had a mortality disadvantage of 4% (HR: 1.04, CI: 0.99–1.09), and women with two migrating parents had a mortality disadvantage of 8% (HR: 1.08, CI: 1.03–1.13). These effects are similar after controlling for unobserved familial effects in tables A1 and A2 or for interactions between the individual migration status and the familial background of migration in table A6.

The adverse effects of parental migration for women, again, indicate that female family members of migrants are disadvantaged rather than advantaged in later life.

## 4 Discussion

In this paper, we have shown that although migrants tend to outlive their non-migrating peers, siblings and children of migrants have no survival advantage after age 50. For men there was no association between familial histories of migration and survival in later life, while women with a migrating sibling or parent had higher mortality rates, instead of the expected lower mortality rates after age 50.

Our findings suggest that the “migrant mortality advantage” is most likely a selection effect across rather than within families. Multiple studies have shown that the decision to migrate is affected by a wide range of individual and familial characteristics (see e.g. Bras, 2002; Bras & Van Tilburg, 2007; Kok et al., 2014; Oris & Alter, 2001; Wintle, 1992). We found, conversely, that the decision of individuals to migrate says little about the health of their family members. Most likely, personality, health, and other individual factors play a large role in the decision to migrate. Therefore, it is surprising that previous studies found that the healthy migrant effect dwindles over time (Kesztenbaum & Rosenthal, 2011; Markides & Rote, 2019) or in case of rural-to-urban migration even (temporary) reverses in times of epidemics (Oris & Alter, 2001; Puschmann et al., 2016). Within a few years after arriving in a new context, the mortality advantage of migrants starts to resemble the receiving society’s mean lifespan, meaning that the survival chances of migrants and non-migrants start to converge. Yet, we found a stable effect of individual migration on later-life mortality.

Although the migrant mortality advantage was not passed on to offspring or shared among siblings in later life, we nevertheless found effects of familial migration histories on female survival in later life. Contrary to our expectations, women with migrating siblings or parents had higher mortality risks in later life than women without migrating siblings or parents. This negative survival effect is unlikely to be rooted in environmental effects, as it affects only women and we found no regional effects. The proportional effects show that the negative effect of family migration were present from age 50 to 100. Most likely, the migration of parents or siblings resulted in a smaller familial support network. During the period of observation female labor opportunities also decreased due to economic downturn and the rise of the male breadwinner model (Janssens, 2020; Timm & Sanborn, 2016), which gave women fewer opportunities to gather income and made them more dependent on family members. Under those circumstances physical distance from family members could be detrimental to female health and survival, as contact between siblings decreased with physical distance (see e.g. Bras & Van Tilburg, 2007). A study on Zeeland by Bras (2002, p. 51–52) also showed that intergenerational income transfers from children to parents were related to proximity. Similar effect might have been present for material and immaterial support by siblings.

As women had less agency in the public sphere, they might have been bereaved of vital sources of support in later life, when their parents and/or siblings moved away from them. The positive self-selection of migrants (see e.g. Borjas et al., 2019) probably made the lives of the female stayers even harder, as they lost vital sources of income in their network. There were also few opportunities for women to move to as, especially in more rural areas with few economic niches, gender norms were adverse to women as there were little opportunities for women to earn income (Humphries, 1991; McNay et al., 2005). This could result in unequal access to food at the dinner table and fewer opportunities to attain income for women from already deprived backgrounds. Although it is mere speculation, female agency might also explain why the female migrant mortality advantage is smaller than the male migrant mortality advantage. Generally, it was the husband who moved to opportunity, rather than the wife. Therefore, gender roles in historical Zeeland might have caused for stronger selection effects of migration on survival after age 50 for men than women.

Our results are based on intraregional migration. This way, we implicitly considered alternative explanations for the healthy migrant effect, as migrants were unable to enjoy temporary benefits of changes in their living environment and diet. Earlier authors have argued that beneficial effects of migration from MENA to Europe or Mexico to the USA might have been beneficial, as individuals move to a wealthier environment without being at risk of man-made diseases that are associated with welfare, for example cardiovascular diseases, aneurisms, and cancer (see e.g. Brussaard et al., 2001; Darmon & Khlat, 2001; Landman & Cruickshank, 2001). After settling and sticking to a new and unhealthier diet this effect might dissipate over time, due to bad housing conditions, heavy labor, or prolonged periods of overnutrition. However, our study shows that the healthy migrant advantage also occurs without changes in the living environment or diet.

Our focus on migration also means that it is unclear whether moving within and between provinces had a similar effect on their families. In our study, we only looked at intra-regional migration and theoretically it is possible that siblings of individuals who migrated to other Dutch provinces or even further away also had a survival advantage. Although we would have of course preferred to have the data on the additional migrants, we nevertheless feel that our results are robust. Firstly, we know that individuals who migrated within Zeeland or to another province had a similar survival advantage (Van den Berg et al., 2020), meaning that effects of individual migration are estimated correctly. Secondly, the familial estimates were all highly robust and none pointed towards a positive association between health and migration. Therefore, it is unlikely that the results were biased by our focus on parents and siblings who lived their whole life within the Dutch province of Zeeland.

All in all, the healthy migrant effect seems to be a strong selection effect. One key to understanding the mechanism is studying why migrants have a survival advantage over the sending and receiving native populations. We showed that survival advantages occur regardless of changes in the living environment and were not caused by the selection of healthy families, rather, female siblings or daughters of migrants had a survival disadvantage. This indicates that unhealthy individuals are less likely to migrate, but that somehow gender negatively affects the later-life survival of a migrant’s female family members, regardless of whether the woman herself migrates. More studies on the family networks of migrants are required to understand whether this pattern can also be observed outside of Zeeland and uncover the mechanism behind it.

Another key to understanding the mechanism behind the healthy migrant effect is in studying how the migrant mortality advantage disappears over time. Our study showed that the migrant mortality advantage also occurs when individuals migrate within the same country and cultural region. This effect is still visible at advanced ages, and our Cox regression models show that the survival advantage is proportional between ages 50 and 100. This finding is at odds with other studies that show that the migrant mortality advantage disappears over time (Kesztenbaum & Rosenthal, 2011; Markides & Rote, 2019; Oris & Alter, 2001; Puschmann et al., 2016; Rosenthal 2011). In this regard it is especially interesting to study whether migrants and stayers differed in their causes of death.

## CRediT author statement

Rick J. Mourits: Conceptualization, Methodology, Formal analysis, Data curation, Writing – Original draft, Writing – Review & Editing, Paul Puschmann: Conceptualization, Methodology, Writing, Writing – Original draft, Writing – Review & Editing.

## Ethical statement

None.

## Declaration of competing interest

None.

## Data Availability

Data is freely accessible at: https://hdl.handle.net/10622/QUJNCD
