# Supplementary material for: Exploring familial factors in the migrant mortality advantage among domestic migrants in later life: Zeeland, the Netherlands, 1812–1962
Source: SSM Popul Health. 2023 Feb 9;22:101359. doi: 10.1016/j.ssmph.2023.101359 (PMC9996346; doi:10.1016/j.ssmph.2023.101359)
Supplement: Multimedia component 1 [file mmc1.docx]

**A1. Stayers and migrants separately**

### *Table A1: Association between migration histories and later-life survival, men*

|  | **1. Migration ego** | | **2. Sibling migration** | | **3. Parental migration** | |
| --- | --- | --- | --- | --- | --- | --- |
|  | **HR + 95% CI** | **p-value** | **HR + 95% CI** | **p-value** | **HR + 95% CI** | **p-value** |
| **Migration** |  |  |  |  |  |  |
| Ego migrated |  |  |  |  |  |  |
| - *No* | ref. | ref. | ref. | ref. | ref. | ref. |
| - *Yes* | **0.91 (0.88-0.95)** | **<0.001** | **0.92 (0.89-0.96)** | **<0.001** | **0.91 (0.88-0.95)** | **<0.001** |
| Sibling migration |  |  |  |  |  |  |
| - *0* | - | - | ref. | ref. | - | - |
| - *1* | - | - | 1.01 (0.96-1.07) | 0.608 | - | - |
| - *2* | - | - | 0.95 (0.90-1.01) | 0.112 | - | - |
| - *3* | - | - | 0.95 (0.89-1.02) | 0.193 | - | - |
| - *4* | - | - | 0.97 (0.89-1.06) | 0.504 | - | - |
| - *5* | - | - | 0.94 (0.84-1.06) | 0.345 | - | - |
| - *6+* | - | - | 0.97 (0.86-1.09) | 0.584 | - | - |
| Parental migration |  |  |  |  |  |  |
| - *None migrated* | - | - | - | - | ref. | ref. |
| - *Father migrated* | - | - | - | - | 1.02 (0.97-1.09) | 0.415 |
| - *Mother migrated* | - | - | - | - | 0.99 (0.94-1.04) | 0.686 |
| - *Both parents migrated* | - | - | - | - | 1.02 (0.97-1.08) | 0.431 |
|  |  |  |  |  |  |  |
| **Control variables** |  |  |  |  |  |  |
| Year of birth | **1.00 (0.99-1.00)** | **<0.001** | **0.99 (0.99-1.00)** | **<0.001** | **0.99 (0.99-1.00)** | **<0.001** |
| Sibship size |  |  |  |  |  |  |
| - *2-4* | ref. | ref. | ref. | ref. | ref. | ref. |
| - *5-8* | 1.02 (0.96-1.10) | 0.492 | 1.04 (0.97-1.11) | 0.308 | 1.02 (0.96-1.09) | 0.500 |
| - *9+* | 1.05 (0.98-1.12) | 0.184 | 1.07 (1.00-1.14) | 0.067 | 1.05 (0.98-1.12) | 0.189 |
| Social class |  |  |  |  |  |  |
| - *Elite* | **1.21 (1.06-1.39)** | **0.005** | **1.21 (1.06-1.39)** | **0.005** | **1.21 (1.06-1.39)** | **0.005** |
| - *Middle class* | 0.99 (0.92-1.07) | 0.853 | 0.99 (0.92-1.07) | 0.834 | 0.99 (0.92-1.07) | 0.852 |
| - *Skilled laborers* | 0.95 (0.90-1.02) | 0.139 | 0.95 (0.90-1.02) | 0.138 | 0.95 (0.90-1.02) | 0.147 |
| - *Laborers* | ref. | ref. | ref. | ref. | ref. | ref. |
| - *Farmers* | **0.89 (0.83-0.95)** | **<0.001** | **0.89 (0.84-0.96)** | **<0.001** | **0.89 (0.83-0.95)** | **<0.001** |
| - *Farm workers* | 0.94 (0.89-1.01) | 0.078 | 0.95 (0.89-1.01) | 0.094 | 0.94 (0.89-1.01) | 0.078 |
| - *Unknown* | **1.08 (1.02-1.16)** | **0.016** | **1.08 (1.02-1.16)** | **0.014** | **1.08 (1.02-1.16)** | **0.016** |
|  |  |  |  |  |  |  |
| **Environment** |  |  |  |  |  |  |
| Child mortality rate | 0.99 (0.97-1.01) | 0.282 | 0.99 (0.97-1.01) | 0.288 | 0.99 (0.97-1.01) | 0.276 |
| Number of inhabitants | 1.02 (0.98-1.06) | 0.384 | 1.01 (0.97-1.06) | 0.506 | 1.02 (0.98-1.06) | 0.372 |
| Net migration rate | 0.99 (0.95-1.03) | 0.579 | 0.99 (0.95-1.03) | 0.575 | 0.99 (0.95-1.03) | 0.577 |
| Island of birth |  |  |  |  |  |  |
| - *Noord-Beveland* | ref. | ref. | ref. | ref. | ref. | ref. |
| - *Schouwen-Duiveland* | 0.98 (0.88-1.10) | 0.766 | 0.99 (0.88-1.11) | 0.834 | 0.98 (0.88-1.10) | 0.769 |
| - *Sint Philipsland* | 0.79 (0.62-1.01) | 0.061 | 0.79 (0.62-1.01) | 0.062 | 0.80 (0.62-1.02) | 0.067 |
| - *Tholen* | 0.90 (0.80-1.01) | 0.061 | 0.90 (0.80-1.01) | 0.062 | 0.90 (0.80-1.01) | 0.065 |
| - *Walcheren* | 0.99 (0.89-1.10) | 0.823 | 1.00 (0.90-1.11) | 0.959 | 0.99 (0.89-1.10) | 0.813 |
| - *Zeeuws Vlaanderen* | 0.99 (0.89-1.10) | 0.868 | 1.00 (0.90-1.10) | 0.948 | 0.99 (0.89-1.10) | 0.853 |
| - *Zuid-Beveland* | 1.06 (0.95-1.17) | 0.303 | 1.08 (0.96-1.18) | 0.271 | 1.05 (0.95-1.17) | 0.322 |

### *Table A2: Association between migration histories and later-life survival, women*

|  | **1. Migration ego** | | **2. Sibling migration** | | **3. Parental migration** | |
| --- | --- | --- | --- | --- | --- | --- |
|  | **HR + 95% CI** | **p-value** | **HR + 95% CI** | **p-value** | **HR + 95% CI** | **p-value** |
| **Migration** |  |  |  |  |  |  |
| Ego migrated |  |  |  |  |  |  |
| - *No* | ref. | ref. | ref. | ref. | ref. | ref. |
| - *Yes* | **0.92 (0.89-0.96)** | **<0.001** | **0.91 (0.87-0.95)** | **<0.001** | **0.91 (0.88-0.95)** | **<0.001** |
| Sibling migration |  |  |  |  |  |  |
| - *0* | - | - | ref. | ref. | - | - |
| - *1* | - | - | **1.09 (1.03-1.15)** | **0.002** | - | - |
| - *2* | - | - | **1.08 (1.02-1.15)** | **0.015** | - | - |
| - *3* | - | - | **1.12 (1.04-1.20)** | **0.004** | - | - |
| - *4* | - | - | 1.07 (0.98-1.17) | 0.146 | - | - |
| - *5* | - | - | **1.15 (1.01-1.30)** | **0.032** | - | - |
| - *6+* | - | - | 1.11 (0.98-1.26) | 0.088 | - | - |
| Parental migration |  |  |  |  |  |  |
| - *None migrated* | - | - | - | - | ref. | ref. |
| - *Father migrated* | - | - | - | - | 1.06 (1.00-1.13) | 0.053 |
| - *Mother migrated* | - | - | - | - | 1.05 (0.99-1.11) | 0.090 |
| - *Both parents migrated* | - | - | - | - | **1.08 (1.02-1.15)** | **0.006** |
|  |  |  |  |  |  |  |
| **Control variables** |  |  |  |  |  |  |
| Year of birth | **1.00 (1.00-1.00)** | **<0.001** | **1.00 (1.00-1.00)** | **<0.001** | **1.00 (1.00-1.00)** | **<0.001** |
| Sibship size |  |  |  |  |  |  |
| - *2-4* | ref. | ref. | ref. | ref. | ref. | ref. |
| - *5-8* | 0.98 (0.91-1.05) | 0.598 | 0.97 (0.90-1.04) | 0.384 | 0.98 (0.92-1.06) | 0.636 |
| - *9+* | 1.02 (0.95-1.10) | 0.546 | 1.00 (0.93-1.07) | 0.946 | 1.03 (0.96-1.10) | 0.477 |
| Social class |  |  |  |  |  |  |
| - *Elite* | 0.89 (0.63-1.27) | 0.529 | 0.90 (0.63-1.27) | 0.534 | 0.90 (0.63-1.27) | 0.552 |
| - *Middle class* | 1.00 (0.88-1.15) | 0.962 | 1.00 (0.88-1.15) | 0.980 | 1.00 (0.87-1.14) | 0.992 |
| - *Skilled laborers* | 0.89 (0.78-1.02) | 0.092 | 0.90 (0.79-1.03) | 0.131 | 0.90 (0.78-1.02) | 0.108 |
| - *Laborers* | ref. | ref. | ref. | ref. | ref. | ref. |
| - *Farmers* | 1.00 (0.92-1.11) | 0.822 | 1.00 (0.92-1.10) | 0.952 | 1.01 (0.92-1.11) | 0.834 |
| - *Farm workers* | 1.01 (0.90-1.11) | 0.964 | 1.00 (0.90-1.10) | 0.932 | 1.00 (0.90-1.11) | 0.984 |
| - *Unknown* | 1.01 (0.96-1.05) | 0.759 | 1.01 (0.96-1.05) | 0.825 | 1.01 (0.96-1.05) | 0.763 |
|  |  |  |  |  |  |  |
| **Environment** |  |  |  |  |  |  |
| Child mortality rate | 1.00 (0.98-1.02) | 0.766 | 1.00 (0.98-1.02) | 0.798 | 1.00 (0.98-1.02) | 0.773 |
| Number of inhabitants | 1.03 (0.99-1.08) | 0.157 | 1.04 (1.00-1.09) | 0.057 | 1.04 (0.99-1.08) | 0.121 |
| Net migration rate | 1.03 (0.99-1.08) | 0.128 | 1.04 (0.99-1.08) | 0.114 | 1.03 (0.99-1.08) | 0.136 |
| Island of birth |  |  |  |  |  |  |
| - *Noord-Beveland* | ref. | ref. | ref. | ref. | ref. | ref. |
| - *Schouwen-Duiveland* | 0.98 (0.86-1.10) | 0.706 | 0.98 (0.86-1.10) | 0.703 | 0.97 (0.86-1.09) | 0.595 |
| - *Sint Philipsland* | 0.79 (0.60-1.04) | 0.090 | 0.80 (0.61-1.05) | 0.111 | 0.80 (0.60-1.05) | 0.104 |
| - *Tholen* | 0.99 (0.88-1.13) | 0.911 | 0.99 (0.88-1.13) | 0.912 | 0.99 (0.88-1.12) | 0.906 |
| - *Walcheren* | 1.06 (0.95-1.19) | 0.294 | 1.05 (0.94-1.17) | 0.422 | 1.05 (0.94-1.17) | 0.394 |
| - *Zeeuws Vlaanderen* | 1.03 (0.92-1.15) | 0.633 | 1.02 (0.91-1.14) | 0.719 | 1.02 (0.92-1.14) | 0.708 |
| - *Zuid-Beveland* | 1.09 (0.97-1.21) | 0.152 | 1.08 (0.96-1.20) | 0.198 | 1.07 (0.95-1.20) | 0.246 |

**A2. Stayers and migrants separately**

### *Table A3: Association between sibling migration and later-life survival, men*

|  | **All men** | | **Stayers** | | **Migrants** | |
| --- | --- | --- | --- | --- | --- | --- |
|  | **HR + 95% CI** | **p-value** | **HR + 95% CI** | **p-value** | **HR + 95% CI** | **p-value** |
| **Migration** |  |  |  |  |  |  |
| Ego migrated |  |  |  |  |  |  |
| - *No* | ref. | ref. | - | - | - | - |
| - *Yes* | **0.93 (0.89-0.97)** | **<0.001** | - | - | - | - |
| Sibling migration |  |  |  |  |  |  |
| - *0* | ref. | ref. | ref. | ref. | ref. | ref. |
| - *1* | 1.01 (0.96-1.06) | 0.706 | 0.99 (0.93-1.04) | 0.619 | 1.06 (0.97-1.15) | 0.220 |
| - *2* | 0.96 (0.91-1.01) | 0.118 | 0.95 (0.88-1.02) | 0.129 | 0.98 (0.89-1.07) | 0.629 |
| - *3* | 0.96 (0.90-1.02) | 0.213 | 0.96 (0.87-1.05) | 0.343 | 0.97 (0.88-1.08) | 0.606 |
| - *4* | 0.97 (0.89-1.05) | 0.406 | 0.99 (0.88-1.12) | 0.860 | 0.97 (0.87-1.09) | 0.621 |
| - *5* | 0.96 (0.86-1.06) | 0.416 | 0.82 (0.68-1.00) | 0.050 | 1.04 (0.91-1.20) | 0.541 |
| - *6+* | 0.98 (0.89-1.09) | 0.765 | 1.07 (0.87-1.30) | 0.525 | 0.99 (0.87-1.12) | 0.826 |
|  |  |  |  |  |  |  |
| **Control variables** |  |  |  |  |  |  |
| Year of birth* | **0.95 (0.94-0.96)** | **<0.001** | **0.95 (0.94-0.97)** | **<0.001** | **0.95 (0.93-0.96)** | **<0.001** |
| Sibship size |  |  |  |  |  |  |
| - *2-4* | ref. | ref. | ref. | ref. | ref. | ref. |
| - *5-8* | 1.03 (0.97-1.10) | 0.306 | 1.05 (0.96-1.14) | 0.280 | 1.02 (0.93-1.12) | 0.683 |
| - *9+* | 1.06 (0.99-1.13) | 0.078 | 1.08 (0.99-1.17) | 0.069 | 1.04 (0.94-1.14) | 0.479 |
| Social class |  |  |  |  |  |  |
| - *Elite* | **1.20 (1.05-1.36)** | **0.005** | 1.19 (0.98-1.44) | 0.080 | **1.21 (1.02-1.44)** | **0.029** |
| - *Middle class* | 1.00 (0.94-1.07) | 0.999 | 0.97 (0.89-1.06) | 0.507 | 1.04 (0.94-1.16) | 0.433 |
| - *Skilled laborers* | 0.96 (0.91-1.02) | 0.157 | 0.94 (0.87-1.01) | 0.081 | 0.99 (0.90-1.09) | 0.874 |
| - *Laborers* | ref. | ref. | ref. | ref. | ref. | ref. |
| - *Farmers* | **0.91 (0.85-0.96)** | **0.002** | 0.94 (0.86-1.02) | 0.126 | **0.88 (0.80-0.97)** | **0.011** |
| - *Farm workers* | 0.95 (0.90-1.01) | 0.107 | **0.92 (0.95-1.00)** | **0.042** | 0.99 (0.90-1.08) | 0.804 |
| - *Unknown* | **1.08 (1.02-1.15)** | **0.009** | 1.07 (1.00-1.16) | 0.065 | 1.10 (0.99-1.22) | 0.073 |
|  |  |  |  |  |  |  |
| **Environment** |  |  |  |  |  |  |
| Child mortality rate | 0.99 (0.97-1.01) | 0.302 | 0.99 (0.96-1.01) | 0.261 | 1.00 (0.97-1.03) | 0.842 |
| Number of inhabitants | 1.01 (0.97-1.05) | 0.645 | 1.01 (0.96-1.07) | 0.715 | 1.01 (0.96-1.06) | 0.645 |
| Net migration rate | 0.98 (0.95-1.02) | 0.408 | 0.97 (0.92-1.02) | 0.210 | 1.01 (0.96-1.06) | 0.721 |
| Island of birth |  |  |  |  |  |  |
| - *Noord-Beveland* | ref. | ref. | ref. | ref. | ref. | ref. |
| - *Schouwen-Duiveland* | 0.99 (0.89-1.09) | 0.807 | 1.00 (0.88-1.14) | 0.975 | 0.95 (0.81-1.12) | 0.562 |
| - *Sint Philipsland* | 0.81 (0.66-1.01) | 0.060 | 0.86 (0.66-1.10) | 0.227 | 0.75 (0.50-1.13) | 0.169 |
| - *Tholen* | 0.91 (0.82-1.01) | 0.067 | 0.92 (0.81-1.05) | 0.222 | 0.89 (0.75-1.05) | 0.174 |
| - *Walcheren* | 1.01 (0.92-1.10) | 0.911 | 1.01 (0.89-1.14) | 0.914 | 0.98 (0.84-1.14) | 0.753 |
| - *Zeeuws Vlaanderen* | 1.01 (0.92-1.10) | 0.090 | 1.07 (0.95-1.20) | 0.259 | 0.92 (0.79-1.07) | 0.284 |
| - *Zuid-Beveland* | 1.06 (0.97-1.17) | 0.210 | 1.10 (0.98-1.24) | 0.110 | 1.00 (0.86-1.17) | 0.968 |

* The survival advantage for year of birth decreased with age from 0.91 (CI: 0.88-0.94) to 0.94 (CI: 0.91-0.96), 0.95 (CI: 0.93-0.97), 0.98 (CI: 0.96-1.01), and 0.95 (CI: 0.90-1.02).

### *Table A4: Association between sibling migration and later-life survival, women*

|  | **All women** | | **Stayers** | | **Migrants** | |
| --- | --- | --- | --- | --- | --- | --- |
|  | **HR + 95% CI** | **p-value** | **HR + 95% CI** | **p-value** | **HR + 95% CI** | **p-value** |
| **Migration** |  |  |  |  |  |  |
| Ego migrated |  |  |  |  |  |  |
| - *No* | ref. | ref. | - | - | - | - |
| - *Yes** | **0.92 (0.89-0.96)** | **<0.001** | - | - | - | - |
| Sibling migration |  |  |  |  |  |  |
| - *0* | ref. | ref. | ref. | ref. | ref. | ref. |
| - *1* | **1.08 (1.03-1.14)** | **<0.001** | **1.10 (1.03-1.17)** | **0.003** | 1.06 (0.98-1.15) | 0.172 |
| - *2* | **1.08 (1.02-1.14)** | **0.009** | 1.08 (1.00-1.16) | 0.061 | 1.07 (0.98-1.16) | 0.138 |
| - *3* | **1.12 (1.05-1.19)** | **<0.001** | 1.07 (0.97-1.19) | 0.174 | **1.13 (1.03-1.23)** | **0.010** |
| - *4* | **1.08 (1.00-1.17)** | **0.044** | 1.05 (0.93-1.20) | 0.423 | 1.09 (0.98-1.21) | 0.117 |
| - *5* | **1.16 (1.04-1.29)** | **0.007** | 1.13 (0.92-1.38) | 0.250 | **1.16 (1.02-1.33)** | **0.025** |
| - *6+* | **1.11 (1.00-1.22)** | **0.049** | 1.20 (0.96-1.49) | 0.102 | 1.08 (0.96-1.23) | 0.195 |
|  |  |  |  |  |  |  |
| **Control variables** |  |  |  |  |  |  |
| Year of birth*** | **0.98 (0.96-0.99)** | **<0.001** | 0.99 (0.97-1.01) | 0.158 | **0.96 (0.95-0.98)** | **<0.001** |
| Sibship size |  |  |  |  |  |  |
| - *2-4* | ref. | ref. | ref. | ref. | ref. | ref. |
| - *5-8* | 0.97 (0.91-1.03) | 0.334 | 0.99 (0.97-1.01) | 0.158 | 0.95 (0.87-1.04) | 0.242 |
| - *9+* | 0.99 (0.93-1.06) | 0.840 | 1.01 (0.91-1.18) | 0.774 | 0.97 (0.89-1.07) | 0.563 |
| Social class |  |  |  |  |  |  |
| - *Elite* | 0.93 (0.68-1.27) | 0.642 | 1.18 (0.74-1.88) | 0.483 | 0.79 (0.51-1.21) | 0.271 |
| - *Middle class* | 0.98 (0.87-1.11) | 0.784 | 1.03 (0.87-1.23) | 0.721 | 0.94 (0.80-1.12) | 0.490 |
| - *Skilled laborers* | **0.88 (0.78-0.99)** | **0.030** | 0.91 (0.77-1.07) | 0.258 | **0.84 (0.70-1.00)** | **0.048** |
| - *Laborers* | ref. | ref. | ref. | ref. | ref. | ref. |
| - *Farmers* | 0.99 (0.91-1.02) | 0.718 | 0.99 (0.87-1.12) | 0.816 | 0.98 (0.88-1.09) | 0.690 |
| - *Farm workers* | 1.00 (0.91-1.10) | 0.994 | 1.06 (0.91-1.24) | 0.466 | 0.97 (0.86-1.09) | 0.595 |
| - *Unknown* | 1.00 (0.96-1.04) | 0.890 | 1.02 (0.97-1.08) | 0.455 | 0.97 (0.92-1.03) | 0.319 |
|  |  |  |  |  |  |  |
| **Environment** |  |  |  |  |  |  |
| Child mortality rate | 1.00 (0.98-1.02) | 0.983 | 1.01 (0.98-1.03) | 0.518 | 0.99 (0.97-1.02) | 0.506 |
| Number of inhabitants | **1.04 (1.01-1.08)** | **0.025** | 1.03 (0.97-1.09) | 0.366 | **1.05 (1.01-1.10)** | **0.026** |
| Net migration rate | 1.04 (1.00-1.07) | 0.060 | 1.03 (0.97-1.09) | 0.338 | 1.04 (0.99-1.08) | 0.122 |
| Island of birth |  |  |  |  |  |  |
| - *Noord-Beveland* | ref. | ref. | ref. | ref. | ref. | ref. |
| - *Schouwen-Duiveland* | 0.97 (0.87-1.07) | 0.552 | 0.91 (0.79-1.05) | 0.182 | 1.04 (0.89-1.21) | 0.641 |
| - *Sint Philipsland* | **0.79 (0.63-0.99)** | **0.043** | **0.72 (0.55-0.94)** | **0.017** | 1.06 (0.68-1.64) | 0.811 |
| - *Tholen* | 0.98 (0.88-1.08) | 0.643 | 0.95 (0.83-1.10) | 0.510 | 1.00 (0.85-1.18) | 0.986 |
| - *Walcheren* | 1.04 (0.94-1.14) | 0.471 | 1.02 (0.90-1.16) | 0.746 | 1.07 (0.93-1.24) | 0.347 |
| - *Zeeuws Vlaanderen* | 1.01 (0.92-1.11) | 0.791 | 0.97 (0.86-1.10) | 0.679 | 1.07 (0.93-1.23) | 0.377 |
| - *Zuid-Beveland* | 1.06 (0.96-1.16) | 0.255 | 1.02 (0.90-1.16) | 0.710 | 1.10 (0.95-1.28) | 0.182 |

+ Associations with migration decreased from 0.81 (CI: 0.74-0.90) before age 60 to 0.96 (CI: 0.93-1.00) between ages 60-100. The survival advantage for year of birth decreased linearly with age from 0.93 (CI: 0.90-0.96) to 0.96 (CI: 0.93-0.98), 0.98 (CI: 0.96-1.00), 1.00 (CI: 0.98-1.03), and 1.04 (CI: 0.98-1.10).

### *Table A5: Association between parental migration and later-life survival, men*

|  | **HR + 95% CI** | **p-value** | **HR + 95% CI** | **p-value** | **HR + 95% CI** | **p-value** |
| --- | --- | --- | --- | --- | --- | --- |
| **Migration** |  |  |  |  |  |  |
| Ego migrated |  |  |  |  |  |  |
| - *No* | ref. | ref. | - | - | - | - |
| - *Yes* | **0.92 (0.88-0.95)** | **<0.001** | - | - | - | - |
| Parental migration |  |  |  |  |  |  |
| - *None migrated* | ref. | ref. | ref. | ref. | ref. | ref. |
| - *Father migrated* | 1.02 (0.97-1.08) | 0.364 | 1.03 (0.96-1.10) | 0.368 | 1.03 (0.94-1.12) | 0.551 |
| - *Mother migrated* | 0.99 (0.94-1.04) | 0.646 | 0.97 (0.91-1.03) | 0.281 | 1.03 (0.95-1.11) | 0.507 |
| - *Both parents migrated* | 1.02 (0.96-1.07) | 0.425 | 0.98 (0.91-1.05) | 0.508 | 1.07 (0.99-1.15) | 0.081 |
|  |  |  |  |  |  |  |
| **Control variables** |  |  |  |  |  |  |
| Year of birth* | **0.95 (0.94-0.96)** | **<0.001** | **0.96 (0.94-0.97)** | **<0.001** | **0.94 (0.93-0.96)** | **<0.001** |
| Sibship size |  |  |  |  |  |  |
| - *2-4* | ref. | ref. | ref. | ref. | ref. | ref. |
| - *5-8* | 1.02 (0.96-1.09) | 0.497 | 1.03 (0.95-1.12) | 0.413 | 1.00 (0.91-1.10) | 0.969 |
| - *9+* | 1.04 (0.98-1.10) | 0.204 | 1.06 (0.98-1.15) | 0.146 | 1.01 (0.92-1.11) | 0.843 |
| Social class |  |  |  |  |  |  |
| - *Elite* | **1.20 (1.06-1.36)** | **0.005** | 1.19 (0.98-1.44) | 0.081 | **1.22 (1.03-1.44)** | **0.025** |
| - *Middle class* | 1.00 (0.94-1.07) | 0.965 | 0.97 (0.89-1.06) | 0.491 | 1.04 (0.94-1.16) | 0.422 |
| - *Skilled laborers* | 0.96 (0.91-1.02) | 0.170 | 0.94 (0.87-1.01) | 0.085 | 0.99 (0.90-1.09) | 0.885 |
| - *Laborers* | ref. | ref. | ref. | ref. | ref. | ref. |
| - *Farmers** | **0.90 (0.85-0.96)** | **0.001** | 0.94 (0.86-1.02) | 0.114 | **0.88 (0.80-0.97)** | **0.007** |
| - *Farm workers* | 0.95 (0.90-1.01) | 0.090 | **0.92 (0.85-1.00)** | **0.038** | 0.99 (0.90-1.08) | 0.769 |
| - *Unknown** | **1.08 (1.02-1.15)** | **0.010** | 1.07 (1.00-1.16) | 0.058 | 1.09 (0.99-1.21) | 0.090 |
|  |  |  |  |  |  |  |
| **Environment** |  |  |  |  |  |  |
| Child mortality rate | 0.99 (0.97-1.01) | 0.283 | 0.99 (0.96-1.01) | 0.237 | 1.00 (0.97-1.03) | 0.835 |
| Number of inhabitants | 1.01 (0.98-1.05) | 0.501 | 1.02 (0.96-1.07) | 0.545 | 1.02 (0.97-1.07) | 0.481 |
| Net migration rate | 0.98 (0.95-1.02) | 0.408 | 0.97 (0.92-1.02) | 0.247 | 1.01 (0.96-1.06) | 0.725 |
| Island of birth |  |  |  |  |  |  |
| - *Noord-Beveland* | ref. | ref. | ref. | ref. | ref. | ref. |
| - *Schouwen-Duiveland* | 0.98 (0.89-1.11) | 0.734 | 1.00 (0.88-1.14) | 0.962 | 0.94 (0.79-1.10) | 0.436 |
| - *Sint Philipsland* | 0.82 (0.66-1.01) | 0.064 | 0.86 (0.66-1.10) | 0.227 | 0.75 (0.50-1.13) | 0.170 |
| - *Tholen* | 0.91 (0.82-1.01) | 0.068 | 0.92 (0.81-1.05) | 0.218 | 0.88 (0.74-1.05) | 0.156 |
| - *Walcheren* | 1.00 (0.91-1.09) | 0.941 | 1.00 (0.89-1.13) | 0.996 | 0.96 (0.83-1.12) | 0.599 |
| - *Zeeuws Vlaanderen* | 1.00 (0.91-1.09) | 0.984 | 1.06 (0.94-1.19) | 0.325 | 0.92 (0.79-1.06) | 0.249 |
| - *Zuid-Beveland* | 1.06 (0.96-1.16) | 0.258 | 1.10 (0.97-1.24) | 0.133 | 0.99 (0.85-1.15) | 0.896 |

* The survival advantage for year of birth decreased with age from 0.91 (CI: 0.88-0.94) to 0.94 (CI: 0.91-0.96), 0.95 (CI: 0.93-0.97), 0.98 (CI: 0.96-1.01), and 0.95 (CI: 0.90-1.02).

### *Table A6: Association between parental migration and later-life survival, women*

|  | **HR + 95% CI** | **p-value** | **HR + 95% CI** | **p-value** | **HR + 95% CI** | **p-value** |
| --- | --- | --- | --- | --- | --- | --- |
| **Migration** |  |  |  |  |  |  |
| Ego migrated |  |  |  |  |  |  |
| - *No* | ref. | ref. | - | - | - | - |
| - *Yes* | **0.93 (0.90-0.97)** | **<0.001** | - | - | - | - |
| Parental migration |  |  |  |  |  |  |
| - *None migrated* | ref. | ref. | ref. | ref. | ref. | ref. |
| - *Father migrated* | **1.06 (1.01-1.12)** | **0.029** | 1.07 (1.00-1.15) | 0.055 | 1.05 (0.97-1.14) | 0.202 |
| - *Mother migrated* | 1.04 (0.99-1.09) | 0.087 | 1.05 (0.99-1.12) | 0.131 | 1.04 (0.97-1.12) | 0.312 |
| - *Both parents migrated* | **1.08 (1.03-1.13)** | **0.001** | 1.03 (0.96-1.11) | 0.444 | **1.11 (1.04-1.19)** | **<0.001** |
|  |  |  |  |  |  |  |
| **Control variables** |  |  |  |  |  |  |
| Year of birth* | **0.98 (0.96-0.99)** | **<0.001** | 0.99 (0.97-1.01) | 0.222 | **0.96 (0.95-0.98)** | **<0.001** |
| Sibship size |  |  |  |  |  |  |
| - *2-4* | ref. | ref. | ref. | ref. | ref. | ref. |
| - *5-8* | 0.99 (0.93-1.05) | 0.633 | 1.00 (0.92-1.09) | 0.956 | 0.97 (0.89-1.06) | 0.454 |
| - *9+* | 1.02 (0.96-1.09) | 0.436 | 1.04 (0.95-1.13) | 0.390 | 1.01 (0.93-1.10) | 0.850 |
| Social class |  |  |  |  |  |  |
| - *Elite* | 0.93 (0.68-1.28) | 0.656 | 1.19 (0.75-1.89) | 0.465 | 0.78 (0.51-1.20) | 0.263 |
| - *Middle class* | 0.98 (0.87-1.11) | 0.736 | 1.04 (0.87-1.23) | 0.685 | 0.93 (0.78-1.10) | 0.395 |
| - *Skilled laborers* | **0.87 (0.77-0.98)** | **0.023** | 0.91 (0.77-1.07) | 0.239 | **0.83 (0.69-0.99)** | **0.036** |
| - *Laborers* | ref. | ref. | ref. | ref. | ref. | ref. |
| - *Farmers** | 0.99 (0.92-1.08) | 0.858 | 1.00 (0.88-1.13) | 0.988 | 0.98 (0.88-1.09) | 0.719 |
| - *Farm workers* | 1.01 (0.92-1.10) | 0.906 | 1.07 (0.91-1.24) | 0.418 | 0.97 (0.87-1.09) | 0.635 |
| - *Unknown** | 1.00 (0.96-1.04) | 0.969 | 1.02 (0.97-1.08) | 0.392 | 0.97 (0.92-1.03) | 0.351 |
|  |  |  |  |  |  |  |
| **Environment** |  |  |  |  |  |  |
| Child mortality rate | 1.00 (0.98-1.02) | 0.984 | 1.01 (0.98-1.04) | 0.462 | 0.99 (0.96-1.02) | 0.470 |
| Number of inhabitants | 1.03 (1.00-1.07) | 0.076 | 1.01 (0.96-1.07) | 0.642 | **1.05 (1.00-1.10)** | **0.036** |
| Net migration rate | 1.03 (1.00-1.07) | 0.080 | 1.02 (0.97-1.08) | 0.395 | 1.04 (0.99-1.08) | 0.121 |
| Island of birth |  |  |  |  |  |  |
| - *Noord-Beveland* | ref. | ref. | ref. | ref. | ref. | ref. |
| - *Schouwen-Duiveland* | 0.96 (0.86-1.06) | 0.414 | 0.91 (0.79-1.04) | 0.171 | 1.02 (0.87-1.19) | 0.831 |
| - *Sint Philipsland* | **0.79 (0.63-0.99)** | **0.041** | **0.72 (0.55-0.94)** | **0.016** | 1.04 (0.67-1.62) | 0.853 |
| - *Tholen* | 0.97 (0.88-1.08) | 0.630 | 0.96 (0.84-1.10) | 0.550 | 0.99 (0.84-1.17) | 0.917 |
| - *Walcheren* | 1.04 (0.95-1.14) | 0.435 | 1.04 (0.91-1.18) | 0.582 | 1.06 (0.92-1.23) | 0.431 |
| - *Zeeuws Vlaanderen* | 1.01 (0.92-1.11) | 0.781 | 0.98 (0.87-1.10) | 0.731 | 1.06 (0.92-1.23) | 0.425 |
| - *Zuid-Beveland* | 1.05 (0.95-1.15) | 0.330 | 1.03 (0.90-1.16) | 0.701 | 1.09 (0.94-1.26) | 0.269 |

+ Associations with migration decreased from 0.80 (CI: 0.73-0.89) before age 60 to 0.96 (CI: 0.92-0.99) between ages 60-100. The survival advantage for year of birth decreased linearly with age from 0.93 (CI: 0.90-0.96) to 0.96 (CI: 0.93-0.98), 0.98 (CI: 0.96-1.00), 1.00 (CI: 0.98-1.03), and 1.04 (CI: 0.98-1.10).
